# The Mechanisms Through Which Fire Drives Population Change in Terrestrial Biota

**DOI:** 10.1111/gcb.70479

**Published:** 2025-09-22

**Authors:** Ella Plumanns‐Pouton, Julianna L. Santos, Cristina Aponte, Lluís Brotons, Luke T. Kelly, Stephen C. Mason, Kirsten Parris, Lauren Ponisio, David A. Keith

**Affiliations:** ^1^ CREAF Cerdanyola del Vallès Spain; ^2^ The School of Agriculture, Food, and Ecosystem Sciences The University of Melbourne Parkville Victoria Australia; ^3^ Departamento de Dinámica y Gestión Forestal Instituto de Ciencias Forestales (ICIFOR, INIA‐CSIC) Madrid España; ^4^ CSIC Cerdanyola del Vallès Spain; ^5^ CTFC Solsona Spain; ^6^ Department of Natural Sciences Immaculata University Immaculata Pennsylvania USA; ^7^ The School of Biosciences The University of Melbourne Parkville Victoria Australia; ^8^ Institute of Ecology and Evolution, Department of Biology University of Oregon Eugene Oregon USA; ^9^ Centre for Ecosystem Science, Biological, Earth & Environmental Sciences University of New South Wales Kensington New South Wales Australia

**Keywords:** fire regimes, fire‐related mechanisms, fire‐related threats, fire‐related traits, functional traits, population change, population viability, species extinction

## Abstract

Global fire regime change is threatening terrestrial biodiversity. Understanding how these changes affect biota is essential to protect biodiversity now and into the future. A targeted examination of the mechanisms through which fire influences populations will help achieve this by enabling comparisons and connections across taxa. Here, we develop a cross‐taxa framework that identifies mechanisms through which fire regimes influence terrestrial species populations over different time scales, and traits on which those mechanisms depend. We focus on amphibians, birds, fungi, insects, mammals, plants, and reptiles. First, we identify key mechanisms through which fire regimes influence species populations across different taxonomic groups. Second, we link these mechanisms to functional traits that influence the relevance to different species. Third, we identify traits that shape the vulnerability—or conversely, resilience—of species populations to frequent, high‐intensity, and large wildfires that are emerging as a threat in many parts of the world. Finally, we highlight how this integrative framework can be useful for understanding and identifying fire‐related threats common to different taxa across the globe and for guiding future research on fire‐related population change.

## Graphical abstract

Understanding the mechanisms through which fire influences populations is necessary to protect species from fire regime change. We develop a cross‐taxa framework that identifies mechanisms through which fire regimes influence terrestrial species populations, including for amphibians, birds, fungi, insects, mammals, plants, and reptiles. We identify shared traits across taxa that shape the vulnerability—or conversely, resilience—of species populations to different kinds of fire regimes. This integrative framework will help understand and identify fire‐related threats common to different taxa across the globe.

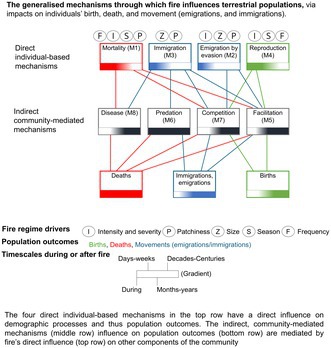

## 1 Introduction

Fire is a fundamental ecological process that shapes biodiversity globally (McLauchlan et al. 2020). Fires can be defined by their intensity, patchiness, and size, as well as their impact on plant biomass—called severity (Krebs et al. 2010). When fire occurs repeatedly over time, it forms a fire regime, which is further characterized by the frequency of fire and seasonal timing (Gill and Allan 2008; Plumanns‐Pouton et al. 2025; Table 1). Many species have evolved fire‐related traits that enable their persistence under particular fire regimes (Pausas 2019; Rainsford et al. 2021). However, fire regimes are changing globally due to climate change, land‐use change, and new patterns of fire suppression, exclusion, and prescription (Abatzoglou et al. 2018; Duane et al. 2021; Kelly et al. 2023). Novel fire regimes threaten terrestrial biota through changes to the frequency, timing, and spatial variation to which species are adapted (de la Barrera et al. 2018; Le Breton et al. 2022; Miller et al. 2019). Research that develops approaches to understand how fire regimes drive population changes is useful for assessing fire‐related threats to biodiversity (Driscoll et al. 2024; Auld et al. 2022).

**TABLE 1:** Key terminology used throughout this review.

Term	Definition
Life cycle	The transition through life stages of an organism, from birth to death.
Demographic process	Processes that shift the size and structure of populations, primarily through births, deaths and movements into and out of those populations (emigration and immigration).
Fire‐related mechanism	The process of change explained by a relationship between a driver and a causal phenomenon—in this context, between fire and biota.
Functional trait	A characteristic (morphological, physiological, phenological or ecological) of an individual, propagule, or other level of biological organization, the expression or value of which influences how an organism responds to and/or behaves within the environment (Fountain‐Jones et al. 2015). Here, we focus on functional traits related to fire‐driven mechanisms of population change.
Fire regime	The patterns of fire within a specific space–time window. The fire regime includes the intensity, severity, patchiness, size and season of fire events and how frequently they occur (Plumanns‐Pouton et al. 2025).
Persistence	At an individual level, the capacity to cope with environmental conditions, continue to live, and perform basic life functions. At a population level, the converse of extinction over a specified time frame.

Integrated, cross‐taxa examination of how fire influences populations is helpful to identify and manage fire‐related threats to species, including those that arise from species interactions. A cross‐taxa approach can complement species‐specific strategies and is essential for understanding context‐dependent responses to fire (Kelly et al. 2018) and identifying key functional traits at the species level (Kattge et al. 2020). A large body of work has demonstrated many ways through which fire influences species within singular taxonomic groups (e.g., Andersen et al. 2014; Beale et al. 2018; Carbone et al. 2019; Farnsworth et al. 2014; Penman et al. 2015). The focus on specific taxa within fire ecology is understandable, given the diversity of life cycles and functional traits (Table 1) across major taxonomic groups and the concentrated resources, skills, and expertise typically available for studies. Consequently, understanding how fire drives biotic change through interactions with demographic processes (Table 1) is more developed for some taxonomic groups, such as plants (Auld et al. 2022), than for others, such as amphibians (dos Anjos et al. 2021) and most invertebrates (Saunders et al. 2021).

As the knowledge base grows, it becomes clear that various fire‐related mechanisms can impact the population dynamics of different species under different fire regimes, leading to population stability, increases, or declines and extirpations (DAWE 2022). We therefore require synthesis of fire‐related population change related to species from a broader range of taxonomic groups. This will facilitate a better understanding of fire‐related threats to biota and the mechanisms and traits that mediate this, and inform pathways to cross‐taxa fire management that benefit the full diversity of life. This is important because species coexist in ecosystems, and their interactions directly influence their population outcomes. An integrated approach will thus enable the identification of trade‐offs between species that exhibit divergent responses and facilitate prioritization. Further, by understanding how fire‐related population change is shared across taxa, we can potentially leverage knowledge from species in one taxon and apply it to functionally comparable species from other taxa. In this review, we ask: (1) Are there general ecological mechanisms regulating how populations respond to fire regimes that apply across a broad range of taxa? If so, what are they? And (2) Are there functional traits shared across species within different taxonomic groups that make them more vulnerable, or resilient, to specific components of fire regimes?

Understanding the impact of fire on species from several taxa involves synthesizing the mechanisms of fire‐related changes to populations and the relevant functional traits that enable these changes. Multiple frameworks have attempted to do this for specific taxa. For example, the Vital Attributes framework predicts the persistence of plant populations under recurring fires based on selected functional traits that different species possess, pertaining to survival, propagule arrival, establishment pattern, and timing of the life cycle (Noble and Gitay 1996; Noble and Slatyer 1980). More recent research has further demonstrated the effectiveness of using plant demographic processes and traits in successfully predicting fire‐related changes (Keith et al. 2007; Plumanns‐Pouton, Swan, et al. 2024a). Frameworks of demographic change have also been developed for reptiles (Santos, Sitters, et al. 2022) and mammals (Santos, Hradsky, et al. 2022). However, a more integrative framework is needed to incorporate a wider range of terrestrial biota and to identify fundamental mechanisms of ecological change that potentially apply to species with different forms and functions.

**FIGURE 1:**
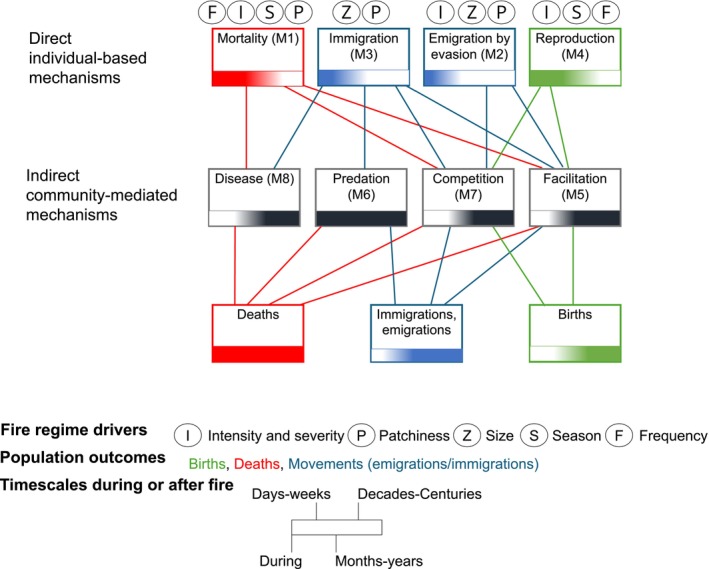
A proposed framework for the generalized mechanisms through which fire influences terrestrial populations. We show eight proposed key mechanisms through which fire influences population change in terrestrial biota via impacts on individuals' birth, death, and movement (emigrations and immigrations). The four direct individual‐based mechanisms in the top row have a direct influence on demographic processes and thus population outcomes and operate during, soon after, or several years after fire. The indirect, community‐mediated mechanisms (middle row) influence on population outcomes (bottom row) are mediated by fire's direct influence (top row) on other components of the community and operate during fire or from days, to weeks to centuries after fire, depending on community structure and characteristics of ecosystems. Population outcomes related to births are colored in green, deaths in red, and movements (emigrations and immigrations) in blue. Examples of important fire regime attributes that commonly influence each direct, individual‐based mechanism are shown next to each mechanism. The timescales at which each mechanism and population outcome can occur over is represented by a gradient scale, where the darker colours indicate it applies at such a timescale. The timescale movies from during the fire, to days to weeks after the fire, to months to years after the fire, to decades to centuries after the fire, in equal quarters of the scale.

Here, we propose a generalized framework outlining the mechanisms by which fire regimes (Figure 1) influence terrestrial species populations (Figure 1). Our focus is on the biotic, demographic processes through which fire affects species—either directly or via trophic interactions. While fire also alters ecosystem‐scale processes, we aim to advance generalizable knowledge across biota by integrating insights across taxonomic groups. This knowledge could provide a foundation for any future ecosystem‐level synthesis. We draw on our expertise in specific taxa, alongside a targeted literature review, to develop a framework relevant to amphibians, birds, fungi, insects, mammals, plants, and reptiles. This allows us to describe key pathways through which fire drives species population changes in a shared way within these groups. Although research on fire impacts on other invertebrates—such as spiders and springtails—is increasing (McLean et al. 2023; Santorufo et al. 2024), we focus on insects, which dominate fire‐related invertebrate studies (Saunders et al. 2021). We then synthesize literature to group functional traits linked to these mechanisms, providing a foundation for identifying fire‐related threats to species and communities. We illustrate the framework using an exemplar fire regime—characterized by high frequency, high intensity, and spatially uniform fires—highlighting key traits associated with species vulnerability and resilience. For each trait, we provide examples from diverse taxonomic groups across global regions. Finally, we examine additional interacting threats that mediate fire's impact on populations and outline opportunities for future research and conservation action.

## 2 Key Mechanisms of Fire‐Related Change in Populations

We propose eight key mechanisms through which fire regimes influence population demographics of terrestrial species. In the first four mechanisms (M), fire directly impacts individuals' birth, death, and emigration/immigration (demographic processes), and thus affects population outcomes (Figure 1). In the last four mechanisms, fire primarily alters birth, death, and/or emigration/immigration indirectly through community‐based interactions (facilitation, predation, competition, disease, and parasitism)—we refer to these as indirect mechanisms (Figure 1). In these four indirect mechanisms, a species' population outcome is mediated by fire's direct influence on the demographic processes of other members of the community and subsequent trophic interactions.

These mechanisms operate across different time scales (Figure 1). While the direct, individual‐based mechanisms operate during or soon after a fire, they may or may not have longer‐term consequences for population change depending on whether fire‐cued or inter‐fire reproduction compensates for fire‐related mortality. Most of the indirect community‐mediated mechanisms occur after fire, sometimes immediately but often in the weeks to decades or centuries after fire, depending on the species and communities they are part of. For example, fire‐promoted predation can occur during a fire, but also fire can influence predation dynamics overall for several decades (Doherty et al. 2022). Fire's influence on the eight mechanisms can be promotive or disruptive (Table S1). For example, fire's direct influence on immigration (M3) during or soon after a fire can be both promotive, encouraging immigration to a burnt place (Evans 1971), or disruptive, preventing immigration to a burnt place (Applestein et al. 2022). Fire's indirect influence on immigration (via the competition, facilitation, and predation dynamics with other taxa) can also be promotive or disruptive. This ultimately depends on the species and, more specifically, on its traits.

**FIGURE 2:**
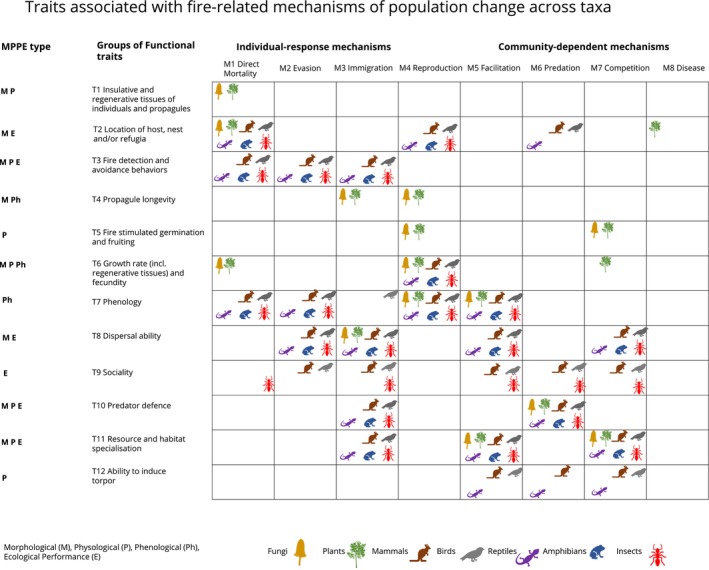
Examples of groups of functional traits that determine the mechanisms of fire‐related change. The columns represent the key mechanisms of population change, and the rows relate the 12 groups of functional traits. The value and expression of these functional traits determine individuals' responses to fire and thus produce the mechanism of change in species populations. Study taxa are represented as colored icons, where the mechanism and associated trait group apply to at least one species in the taxon, according to evidence. The MPPE type demonstrates the kinds of traits—morphological (M), physiological (P), phenological (Ph), and ecological (E) performance traits—that make up the groups of functional traits.

We suggest that different fire regime attributes, such as the intensity, season, size, patchiness of fires, and how frequently they occur, also have different magnitudes of influence on the direct fire‐related mechanisms. The effects of fire regimes on population outcomes can thus happen directly to individuals or via community‐based pathways. For example, a species may not be killed in an intense fire, but their essential food resources may be drastically reduced due to high severity (disruption of facilitation; M5). Dietary shifts in situ may lead to death by starvation, emigration to find new food resources, or a combination of those responses at the population level (Figure 1). Here, we group the mechanisms to enable explanation and comparison across multiple taxonomic groups, but each mechanism does not necessarily apply to all taxa (Figure 2; Tables S1 and S2). When a mechanism applies to a species within a taxonomic group, it depends on the species’ traits (Figure 2).

We explain the mechanisms in detail below:

### 2.1 Direct Mechanisms

#### 2.1.1 Direct Mortality (Vs. Survival)

There is strong evidence of fire directly killing individuals during or shortly after exposure to lethal heat or chemical emissions. Mortality of injured individuals may extend over weeks or, in the case of long‐lived plants, years (Busby et al. 2024; Hood et al. 2018). Higher rates of energy released during combustion typically cause higher mortality of standing plants, fungi, and their seeds or spores (Pérez‐Izquierdo et al. 2021; Reazin et al. 2016; Tangney et al. 2020; Whelan and Ayre 2022). Similarly, high radiant heat, smoke exposure, and/or direct flame consumption can kill animals (Dole et al. 2023; Jolly et al. 2022; Zylstra 2023) and their eggs (Branson and Vermeire 2007; Pilliod et al. 2003; Shine et al. 2016). In contrast, in situ survival depends on heat‐resistant or regenerative organs (plants, fungi) or the avoidance of lethal heat either within the soil profile or via sheltering in micro‐refugia, including underground burrows, tree canopies, plants, and logs during the fire event (all taxa; Robinson et al. 2013; Geluso and Bragg 1986; Long 2009; Vogl 1973).

#### 2.1.2 Fire Evasion Induced Emigration

Animals may emigrate during fire, evading its lethal heat and smoke by fleeing beyond the fire extent (Figure 1; Nimmo et al. 2019; Abrahms et al. 2017), at varying distances via land or flight (Brotons et al. 2012). The legacies of this emigration in populations can persist across multiple generations, facilitating colonization of unburnt areas and potentially affecting gene flow and genetic diversity of populations (Banks et al. 2017; Davies et al. 2016). Sessile organisms, such as plants and fungi, are incapable of evading fire on their own by individual movement, but animals can contribute to seed and spore emigration from within fire‐affected sites to new areas (Moore and Vander Wall 2015; Peterson and Parker 2016), and convection dispersal is plausible during forest fires (Pisaric 2002; Monty et al. 2013; Camacho et al. 2018).

#### 2.1.3 Fire‐Mediated Immigration

During or shortly after a fire, immigration towards fire‐affected areas may occur (Saint‐Germain et al. 2008). Chemical, visual, or auditory fire cues can attract mammals, birds, reptiles, and insects via the “magnet effect” (Archibald et al. 2005; Nimmo et al. 2019), coinciding with the appearance of new dietary and habitat resources (Cherry et al. 2017; Liu et al. 2022). Individuals that immigrate to new areas may successfully establish, reproduce, and thus colonize recently burnt areas, while others may be transient foragers. Fire‐cued immigration is not documented in amphibians. Through endozoochory and exozoochory (animal dispersal), fire can also facilitate the arrival of plant and fungal propagules into burnt areas (Benedicto‐Royuela et al. 2024; Horton 2017), but the role of fire in promoting plant and fungi dispersal is poorly understood (Keith et al. 2020).

Fire can also interrupt immigration during or shortly after a fire, and this effect may be related to the size of the fire's extent, its patchiness, and the broader fire mosaic within a landscape. In the case of plants and fungi, large and uniform fire can limit immigration if fire footprints create long distances to propagule sources relative to dispersal capability (Applestein et al. 2022; Christopoulou et al. 2014; Galante et al. 2011). In the case of animals, fire can disrupt movement pathways due to changes in habitat connectivity (Fordyce et al. 2016). Recently burned areas may be unsuitable for immigration due to the removal of protective habitat, which would prevent free movement, i.e., “landscape of fear” (Gaynor et al. 2019).

#### 2.1.4 Fire‐Mediated Reproduction

During and after a fire, reproduction in plants and fungi may be promoted through fire‐stimulated flowering and fruiting, an effect that may extend for several years (Hughes et al. 2020; Lamont and Downes 2011). Fire is not known to directly promote reproduction in other taxa—even though it can benefit animals by promoting resources that enhance reproduction (Mechanism 5). However, fire can directly disrupt the reproduction of all taxa. For plants, fire frequency can be quicker than the time necessary for new individuals to mature, and for surviving individuals, fruit production can cease for a number of years after fire before recommencing reproduction (Plumanns‐Pouton, Swan, et al. 2024b). Direct disruption of animal reproduction due to the timing of fires is not well documented across many species but has been demonstrated, such as for the Northern Quoll ( *Dasyurus hallucatus* ; Griffiths and Brook 2015) and red‐backed fairy wren (*Malurus melanocephalus*; Murphy et al. 2010). Fire is well known to influence animal reproduction indirectly via reducing the suitability of habitats (M5) or via increasing predation of nests (M6).

### 2.2 Indirect Mechanisms

#### 2.2.1 Fire Mediated Facilitation

Both shortly after fire and persisting across long, decadal time scales, fire can affect access to food and habitat resources for all taxa, and thus indirectly populations' capacity to persist. For plants and fungi, fire can enhance access to resources by altering soil and litter composition, liberating nutrients from biomass, increasing available dead wood (Chungu et al. 2020; Suzuki 2017), and promoting pollinator visitation (Carbone et al. 2024; Nuland et al. 2013). Fire can increase food for animals by facilitating new plant growth (Westlake et al. 2020), increasing dead wood (Jung et al. 2020; Saint‐Germain et al. 2008; Ulyshen 2018), stimulating floral resources (Nuland et al. 2013), fruits, and/or seeds (Tunes et al. 2017), and increasing access to prey (Doherty et al. 2022). Fire can also create new habitat resources, such as tree hollows—used by animals for shelter (Inions et al. 1989), warming of soils (Hossack et al. 2009), or the promotion of open environments suitable for basking (Dovčiak et al. 2013). Better resources can increase survival rates (Schwartz and Franzmann 1991) and promote fecundity in some animal populations (Smith et al. 2012).

Fire can also disrupt facilitation through the modification of vegetation structure required for shelter and nesting, such as the reduction of tree canopies, hollows (Haslem et al. 2012; Wagner et al. 2024), and shrubby vegetation, and the resulting soil erosion and sedimentation of water used by species (Gomez Isaza et al. 2022). Fire may alter plant root architecture required for arbuscular mycorrhizal fungi (Hart et al. 2005; Hartnett et al. 2004), or, conversely remove fungi required for plant growth (Dove and Hart 2017). Fire can also disrupt host‐dependent and mutualistic relationships by reducing the abundance of one partner or limiting access to dietary resources for animal taxa, particularly those with narrow dietary niches (Santos et al. 2014). Fire‐driven decreases in resources can diminish the capacity of some species to reproduce and survive and thus may promote movement to new areas (Figure 1; Banks et al. 2011; Bellia et al. 2011; Griffiths and Brook 2015).

#### 2.2.2 Fire‐Promoted Predation

Fire can reduce survival rates of prey populations by quickly attracting predators or herbivores to more open post‐fire habitats (Hovick et al. 2017; Figure 1). The impacts of fire on predation can occur during the fire itself, but also the predator–prey dynamics may be altered across several decades (Doherty et al. 2022). Fires may result in substantial loss of understory vegetation and other habitat components used for shelter (e.g., logs), leaving animal prey populations vulnerable to increased predator activity (Doherty et al. 2022; Hradsky et al. 2017; Janssen et al. 2007; McGregor et al. 2017; Rocha et al. 2008; Wilgers and Horne 2007). Fire can also increase herbivory pressure on plants and mycophagy pressure on fungi through increased access and attraction to novel food sources, reducing plant and fungi population survival (Donaldson et al. 2018; Giljohann et al. 2017; Luo and Fox 1994; Westlake et al. 2020).

#### 2.2.3 Fire‐Mediated Interspecific Competition

Soon after a fire, interspecific competition between plant species may be released (Keith and Bradstock 1994; Tilman 1994; Wilson and Shay 1990), primarily by liberating resources, such as nutrients, water, and light; reducing competitor density or size; and triggering successional pathways (Grime et al. 1997; Poorter et al. 2023). Fire alters the competition dynamics over several decades to centuries via successional pathways (Noble and Slatyer 1980). Fire may also impact interspecific competition in fungal species by changing temperature, soil composition, and resources (Carlsson et al. 2014; Certini et al. 2021).

For animals, interspecific competition is related to Mechanism 5 (fire‐mediated facilitation), as the competition between species is also related to the scarcity of resources. Furthermore, when the overall habitat becomes less favorable to a species, their share of resources may also be simultaneously reduced by the overall habitat becoming more favorable to a competitor species (habitat accommodation model; Fox 1982). There is some indication that fire can cause competitive release in mammals (Allen et al. 2022; Fox et al. 2003) and insects (Burkle et al. 2019; Kaynas 2017), but in general, fire's effect on interspecific competition in animals, and its relationship with habitat facilitation, is poorly understood.

#### 2.2.4 Fire‐Mediated Susceptibility to Disease and Parasitism

In the long term, fire can increase the severity of disease outbreaks in plant communities, likely due to changes in environmental suitability for the pathogen (e.g., microclimate), in combination with reduced resilience of host individuals (Moore et al. 2014; Parker et al. 2006). The influence of fire on disease in animals is not well understood (Albery et al. 2021), but the relationship is likely to be akin to plants, as poor body condition may increase animal susceptibility to disease (Beldomenico and Begon 2010). In the short term, fire may also disrupt pathogenesis of plants if pathogens have external life stages that can be destroyed as fire passes (Roy et al. 2014). This also applies to parasitism of mammals, amphibians, and reptiles if a free‐living stage in the life cycle of parasites exposes them to lethal heat (Álvarez‐Ruiz, Belliure, Santos, et al. 2021; Donaldson et al. 2023; Gallagher et al. 2022; Kaiser et al. 2021; Scasta 2015). Fire may impact the parasitism of birds in the same way, but this is relatively unknown (Churchwell et al. 2008; Pascoe et al. 2023). Fire‐driven dispersal may also introduce new pathogens, contributing to the spread of diseases (Albery et al. 2021).

## 3 Fire‐Related Traits Across Taxa

The mechanisms of fire‐driven population changes in species are mediated by functional traits (Morgan et al. 2021; Plumanns‐Pouton, Swan, et al. 2024a). Functional traits influence birth, death, and movement—and thus fitness in the context of fire. These traits can be morphological, physiological, phenological, or ecological performance traits (Table 1; Fountain‐Jones et al. 2015). Many of these functional traits are thought to have evolved under selection pressures imposed by a long history of fire (He et al. 2016). However, some traits that confer resilience to particular fire regimes may have evolved independently of fire under different selection pressures, such as drought and herbivory (Pausas and Keeley 2014). For example, some fungi form sclerotia—dense tissues that facilitate survival in extreme temperatures—providing insulative protection from fire (Figure 2, Trait 1). However, this trait likely evolved in the context of a range of environmental pressures (Smith et al. 2015; Willetts 1971). We also interpret fire‐related traits as those that influence community interactions, such as competition, predation, and facilitation, because they influence fitness under recurrent fire. In Figure 2, we list 12 groups of traits that influence if and how fire‐related mechanisms affect species populations. Different species possess different combinations of traits that make them resilient or vulnerable to fire.

We have grouped these traits to enable generalization and comparison across taxa, with some traits applying to species across all taxa, others to species in a few taxa, and some to species in only one taxon (Figure 2; Table S3). We recognize that the ways in which these generic traits are described and applied are specific to particular taxa. For instance, the general trait ‘T8 dispersal ability’ (Figure 2) is associated with distinct morphological characteristics across taxa: in birds, traits such as body mass, wingspan, or Hand Wing Index are closely linked to dispersal potential (Alerstam et al. 2007; Baldwin and Myers 2024), whereas in plants, seed mass and dispersal syndrome are important (Petrocelli et al. 2024). Here, we use general terms (Figure 2) to group and characterize the diversity of these specific traits. We have indicated whether morphological, physiological, phenological, or ecological performance taxa‐specific traits might be relevant for each generic trait. Below, we discuss individual taxa and highlight key examples of how the value and expression of traits determine different species fire‐related population change.

### 3.1 Fungi

For fungi with limited dispersal (Chaudhary et al. 2022), the thermal resistance of individuals or host locations is important to survive fire events (Baar et al. 1999). For example, pyrophilous or phoenicoid species can resist high heat via thick‐walled chlamydospores (fire‐resistant spores) and inhabit shallow soil depths that are exposed to temperatures that kill most of their competitors, which allows them to rapidly colonize a post‐fire niche (Bruns et al. 2020; Vrålstad et al. 1998; Figure 2, Mechanism (M)1, Trait (T)1). Extension of hyphae into subsurface soil horizons (e.g., in association with deeper root tips) can also contribute to survival at high fire intensities, as heat diminishes rapidly with soil depth (Cairney and Bastias 2007; Figure 2, M1, T2).

Fire‐cued germination of spores is an important trait that determines the capacity of fungal species to recolonize post‐fire environments (Figure 2, M4, T5; Bruns et al. 2019; Camacho et al. 2018), as is the use of habitat and nutrient resources (Figure 2, M5, T11). For example, some species can survive with lower available organic matter (Certini et al. 2021), can profit from post‐fire flushes in available nitrogen (Suzuki 2017), or have the capacity to break down hydrophobic waxes and pyrogenic organic matter (Fischer et al. 2021; Steindorff et al. 2021). Some fungi, such as *Ascomycetes* species, are highly plastic in their resource usage and can behave as endomycorrhizal or saprotrophic fungi depending on the availability of saprophytic and mycorrhizal sources of nutrition (Cairney and Bastias 2007; Figure 2, M5, T11). In contrast, other fungal taxa, such as *Trichoderma*, are inhibited by post‐fire chemicals in the environment (Widden and Parkinson 1975; Figure 2, M5, T11).

The speed of growth and reproduction (r/K selection) is another important trait in determining fungal fire responses (Figure 2, M5 T6). Some species, such as fast‐growing saprobic fungi from the genera *Pyronema*, *Penicillium*, and *Aspergillus*, can quickly dominate after 25 days post‐fire (Bruns et al. 2020). Dispersal ability also influences post‐fire responses (Figure 2, M3, T8). Quick growth in combination with wide dispersal of spores, such as for *Eurotiomycetes*, is a key determinant of post‐fire establishment (Whitman et al. 2019). Variation in mycophagy defense (Künzler 2018; T10), phenology (Andrew et al. 2018; Lagomarsino Oneto et al. 2020; T7), and dormancy (Nara 2009; T4) are also likely to impact fungal fire responses.

### 3.2 Plants

In plants, specialized insulative and regenerative tissues, such as thick bark, basal and epicormic resprouting organs, serotinous, and heat‐resistant seed coats, mediate plant and seed survival of fire (Clarke et al. 2013; Pausas 2015; Tada et al. 2024; Figure 2, M1, T1). The capacity for fire‐stimulated germination determines the quantities of seeds that are recruited or remain dormant at different fire intensities (Lamont et al. 2019; T5). Burial of seeds and regenerative organs below the soil surface can limit exposure to lethal heat (Pausas et al. 2018; Figure 2, M1, T2). A lack of resprouting (“obligate seeding”) is a particularly important trait expression that shapes risks to fire‐prone populations (Prior and Bowman 2020; Figure 2, M1, T1).

Important phenological traits for plants, including the time taken to mature and accumulate a seed store (Zedler 1995; Figure 2, M4, T6), and seed longevity (Auld et al. 2000; Figure 2, M4, T4), determine if fire disrupts reproduction (Plumanns‐Pouton et al. 2023). For example, the species *Callitris rhomboideia* takes at least six years to produce its first seed and 40 years to reach peak seedbank accumulation, making it more vulnerable to short fire intervals than a species that reaches maturity and peak seedbank accumulation in just one or two years (Plumanns‐Pouton, Swan, et al. 2024b). The time taken to develop insulative and regenerative tissues is a similarly important trait for resprouters, but it is largely understudied (Fairman et al. 2019; Nolan et al. 2021; M4, T6). Fire‐cued germination and flowering (Bell 1999; Miller et al. 2019; Figure 2, M4, T5) and phenological traits, such as the season of flowering, seasonal dormancy, and pollination (Beck et al. 2023; Ooi 2019; Figure 2, M4, T7), determine how fire season influences plant reproduction.

Dispersal distance and syndrome influence rescue and colonization processes post‐fire (Plumanns‐Pouton, Kasel, et al. 2024; Figure 2, T8). Species that disperse locally, through barochory (gravity), for example, are less able to rely on immigration than species that can travel great distances through zoochory (animal) or through anemochory (wind; M3; Ahler et al. 2023; Arnan et al. 2010). Finally, traits that confer tolerance of herbivores and competitors and enable establishment in a range of successional conditions play important roles in determining if species establish across various fire regimes (Barton and Koricheva 2010; Valladares and Niinemets 2008; Figure 2, M7, T10, T11).

### 3.3 Mammals

Although thermal tolerance varies across species, mammals generally have low thermal tolerance (Erskine and Hutchison 1982; Fristoe et al. 2015; Zylstra 2023). As such, the choice of habitat, such as nesting and sheltering substrates, plays an important role in determining fire survival (Culhane et al. 2022; Figure 2, M1, T2). Fire detection and local avoidance behaviors, such as sheltering in underground burrows, are key to the survival of individuals (Figure 2, M1, T3). Fire detection is also important for determining fire evasion‐driven emigration (Figure 2, M2, T3). Dispersal traits, such as body size and speed, play important roles in whether mammals can evade fire (Pla et al. 2023; Figure 2, M2, T8). For example, koalas ( *Phascolarctos cinereus* ) are slow‐moving species and are highly vulnerable to fire‐related injury or death (Law et al. 2022). Sociality can also play an important role in the evasion of fire (Figure 2, M2, T9) and likely the use of post‐fire resources (Figure 2, M5, T9). Alarm calling is a frequently expressed behavior by social mammal species in the face of danger (Hollén and Radford 2009). For example, savanna chimpanzees ( *Pan troglodytes verus* ) can warn other group members about fire and monitor and determine evasion actions as a group (Pruetz and LaDuke 2010). Dispersal behavior is conversely important in determining immigration to burnt areas (Forney and Peacock 2024; Figure 2, M3, T9), which may have longer‐term consequences on local genetic diversity (Smith et al. 2014).

Fire shapes mammals' resource availability (Santos, Belliure, et al. 2022), dependent on the specialization of a species in their habitat and resource use (Dickman and Happold 2022; Vieira and Briani 2013; Zylinski et al. 2022; Figure 2, M5, T11). For example, arboreal mammals reliant on large trees for shelter are vulnerable to the impacts of crown fire on vegetation structure (Chia et al. 2015). Conversely, species that possess traits that provide advantages for foraging in open habitats may benefit from frequent and severe fire. For instance, the Mexican free‐tailed bat ( *Tadarida brasiliensis* ) demonstrates morphological and call adaptations to exploit resources in recently burnt, open habitats (Blakey et al. 2019). Other mammals can cope with reduced resources in post‐fire environments by reducing metabolic demand through induced torpor (Geiser et al. 2018; Figure 2, M5, T12). For example, the echidna ( *Tachyglossus aculeatus* ) can remain in a reduced energetic state for weeks after a fire (Nowack et al. 2016).

Predation defenses are also important in determining mammal success in post‐fire environments (Nimmo et al. 2019; Figure 2, M6, T10), particularly where predator activity increases after fire and when prey species exhibit high site fidelity (Pla et al. 2024; Menon et al. 2025). Fire may influence mammalian reproduction depending on growth rate and fecundity (Figure 2, M4, T6). Mammals with r‐strategies may exploit recently burnt areas, increasing their fitness and reproductive success to rapid population increases (Tokushima and Jarman 2017). For mammals with K strategies, populations may take longer to recover after fire, especially if they rely on resources reduced by fire (Laurenson 1994; Willson et al. 2021). Phenology may also influence reproduction. For example, species with synchronous annual reproduction may be more vulnerable to negative impacts of fire on reproduction (Begg et al. 1981; Griffiths and Brook 2015; Figure 2, M4, T7).

### 3.4 Birds

Birds also have low thermal tolerance but are relatively mobile through flight and sometimes by foot. Nesting location and material are important traits in determining individual and egg mortality during fire (Shine et al. 2016; Figure 2, M1, T2). Fire detection and avoidance behaviors are also important traits that determine mortality (Figure 2, M1, T3). For example, sedge wrens ( *Cistothorus platensis* ) demonstrate fire avoidance behaviors, flying short distances as fire approaches and eventually seeking shelter in protective vegetation (Engstrom 2010).

Dispersal ability is an important determinant of post‐fire colonization (Figure 2, M3, T8). Species that can travel larger distances are more likely to be early post‐fire colonizers (Franklin et al. 2021). For example, the Western black‐eared wheatear ( *Oenanthe hispanica* ) traveled at least 50 km to colonize a post‐fire landscape, although in more general terms post‐fire immigration is limited by distance (Brotons, Pons, and Herrando 2005). Phenology is also important for species that travel to post‐fire areas (Figure 2, M3, T7). Adults of ortolan bunting ( *Emberiza hortulana* ), for example, can disperse longer distances in the breeding season than juveniles (Dale et al. 2005).

One of the most important ways fire influences birds is access to post‐fire resources. Traits determining resource use, such as dietary and nesting preferences, are key determinants of this mechanism (Rainsford et al. 2023; Figure 2, M5, T11). Plasticity of resource use influences persistence across a range of fire histories (Brotons, Herrando, and Martin 2005). For example, the Northern Bobwhite ( *Colinus virginianus* ) demonstrates the capacity to opportunistically use different available resources as nesting substrates, depending on the fire history and spatial mosaic (Carroll et al. 2017). Diet and habitat specialization also play an important role in immigration to burnt areas (Figure 2, M3, T11). For example, fire often attracts carnivorous bird species, such as Swainson's hawk ( *Buteo swainsoni* ; Hovick et al. 2017) and Black Kite ( *Milvus migrans* ; Bonta et al. 2017), which utilize the open post‐fire environment to seek their prey. Similarly, fire's smoke and pyrogenic updraft may be exploited by insectivorous birds for feeding, as insects are carried into the air (Engstrom 2010). Predator defenses (Lima 2009; 10), torpor (Schleucher 2004; 12), reproductive rate (Woinarski and Recher 1997; T6), and sociality (Downing et al. 2020; T9) are also likely to be important in mediating fire's influence on population persistence.

### 3.5 Reptiles

Generally, reptiles have low dispersal ability in comparison to mammals and birds, although some larger reptile species can disperse large distances (Valentine and Schwarzkopf 2009). This makes local avoidance of lethal heat critical to their survival of fire events (Santos, Belliure, et al. 2022). Nesting and sheltering location and substrate (Figure 2, M1, T2) are important traits determining whether reptiles are killed by fire. For example, species that shelter in ground litter, hollow logs, or in the shrub layer are more likely to die due to direct flame contact or suffocation (Jordaan et al. 2020). Fire detection and avoidance behavior are also crucial for surviving fire, as this induces reptiles to seek in situ refugia within the fire perimeter (Figure 2, M1, T3) or to flee beyond the fire extent to new areas (Figure 2, M2, T3). For example, the Mediterranean lizard Algerian psammodromus (*Psammodromus algirus*) can detect smoke and respond accordingly (Álvarez‐Ruiz, Belliure, and Pausas 2021).

Phenology also influences mortality during fire (Edmonds et al. 2024; Figure 2, M1, T7). For example, ecdysis (skin shedding) affects the ability of fire detection in Eastern diamondback rattlesnakes ( *Crotalus adamanteus* ), resulting in elevated mortality rates if fire passes (Means and Campbell 1981). Similarly, seasonal reproductive behavior can influence mortality rates during fire. For example, some South African fossorial reptiles favor shallow soil for finding mates, increasing their exposure to smoke and heat (Jordaan et al. 2020). Reptiles' predominant reliance on in situ survival suggests that fire's influence on reproduction is important and likely determined by traits related to growth rate and phenology (Shine et al. 2016; Weiss and Brower 2021; Figure 2, M4, T6, T7).

Another critical way that fire influences reptiles is by altering food and habitat resources. Recently burned areas with less ground cover might offer additional habitats for some reptile species by improving opportunities for thermoregulation (Costa et al. 2020; Smith et al. 2012). How fire influences food and habitat resources depends on diet and habitat specialization (Lazzari et al. 2022; Figure 2, M5, T11). For example, the insectivorous Starry‐knob‐tailed gecko ( *Nephrurus stellatus* ) is favored in post‐fire environments due to the greater availability of their preferred food in open environments (Smith 2018). Conversely, for the Southern Mallee ctenotus (*Ctenotus atlas*), severe fires lead to a decline in abundance due to the loss of hummock grass cover, which this species depends on (Driscoll and Henderson 2008). Habitat specialization and dispersal ability are therefore important in determining post‐fire immigration and consequently genetic diversity (Ferreira et al. 2019). Data on brumation and predator avoidance after fire for reptiles is limited, but these may be used as a strategy to cope with a lack of resources or predation risk in post‐fire habitats (Fenner and Bull 2007; Lillywhite et al. 1977; Figure 2, M5, T12, M6, T6, T10).

### 3.6 Amphibians

The impact of fire on amphibians is understudied in comparison to other taxa (Jolly et al. 2022). Likely, many amphibians rely largely on in situ survival through the insulative properties of their habitat and refugia (Figure 2, M1, T2, T3). For example, burrowing frog species evade hot fire temperatures in burrows (Penman et al. 2006; Mahony et al. 2022), and arboreal species may shelter in deep tree hollows (Beranek et al. 2023).

For amphibians that can disperse relatively large distances, an important suite of traits is the capacity to detect and evade fire by emigration (Figure 2, M2, T3). There is evidence that some species can detect fire, such as the West African juvenile reed frog ( *Hyperolius nitidulus* ), which can hear the sound of fire and flee accordingly (Grafe et al. 2002). Dispersal distance, alongside habitat specialization, is also important for determining post‐fire immigration, and thus gene flow and genetic diversity (Potvin et al. 2017; Figure 2, M3, T8, T11). For example, genetic diversity of treefrog species (*Litoria* spp.) declined in response to high‐intensity, uniform fires, and population modelling indicated that a low immigration rate was an important contributing factor to the modelled population collapse of the growling grass frog ( *Litoria raniformis* ; Potvin et al. 2017).

Fire can influence the ability of amphibians to complete their life cycle, depending on traits such as nesting/host location, phenology, and growth rate (Hossack and Pilliod 2011). For example, fire reduces the breeding success of Mediterranean frogs that produce eggs and tadpoles in streams and ponds (Muñoz et al. 2019; Figure 2, M4, T2). Fire can also alter amphibians' habitat resources, such as through sedimentation of waterways and the increase of solar radiation, depending on their habitat specialization (Figure 2, M5, T11). For example, as woodland salamanders (genus *Plethodon*) have a fully terrestrial life cycle, they are more likely to be impacted by changes to microhabitat structure driven by fire frequency (Hromada et al. 2018). Conversely, boreal toads ( *Bufo boreas* ) benefit from the increased temperatures in recently burnt habitats (Hossack et al. 2009). Variation in predatory defenses (Murray et al. 2004; T10) is also likely to influence survival in post‐fire environments.

### 3.7 Insects

Little is known about fire's influence on the myriad of invertebrate species, and most fire ecology knowledge of this broad group relates to insects in particular (Mason et al. 2021). The location, type, and substrate of nests that insects use are important in determining their capacity to survive fire events (Cane and Neff 2011; Jofré et al. 2022; Rhee and Hochkirch 2023; Figure 2, M1, T2). For example, arboreal and epigeic insects are highly vulnerable to fires regardless of their life stage (Frizzo et al. 2012; Thom et al. 2015). There is support, however, that insects that utilize underground burrows and pupate in wood can survive certain intensity fires (Bess et al. 2002; Cane and Neff 2011; DeSouza et al. 2003; New 2014; Selfridge et al. 2019). The capacity to detect and avoid fire also influences whether insects survive fire in situ or evade the fire's extent (Evans 1966; Liu et al. 2022; Figure 2, M1, T3, M2, T3).

Fire detection also influences species immigration to burnt habitats (Bell et al. 2022; Figure 2, M3, T3). For example, over 30 species of pyrophilous insects are known to detect and move towards recently burnt landscapes (Evans 1971; Saint‐Germain et al. 2008). Dispersal traits such as body size and flight influence insects' ability to evade fire (Barber et al. 2017; Driscoll et al. 2020; Mason et al. 2023; Figure 2, M2, T8), and, conversely, colonize recently burnt environments (Koltz et al. 2018; Lazarina et al. 2016; Mason et al. 2023; Figure 2, M3, T8). Sociality traits such as colony type also play a role in immigration and survival (Glasier et al. 2015; Vidal‐Cordero et al. 2023; Figure 2, M3, T9, M1, T9).

Fire can disrupt insect reproduction, dependent on species phenology and growth rate (Figure 2, M4, T6, T7). This includes the timing of reproduction, which can be influenced by the fire season (Decker and Harmon‐Threatt 2019). For example, Cristina's timema ( *Timema cristinae* ) participates in diapause in the dry season, when they are resilient to fire, and produces larvae and nymphs in spring, when they are vulnerable to the passage of fires (Sandoval 2000). Univoltine insects, those that produce just one set of offspring per year, may be particularly vulnerable to frequent or unseasonal fire (Brown et al. 2017).

Fire can influence the availability of dietary and habitat resources of insects, depending on species' diets and resource use (Arnan et al. 2013; Bargmann et al. 2016; Figure 2, M5, T11). For example, fire can increase floral resources (Goldas et al. 2022), promote foraging activity in bumble bees ( *Bombus vosnesenskii* ; Mola and Williams 2018), or increase nesting resources in dead wood or bare ground (Potts et al. 2005). However, the converse is also true. Some insect species have strong host specificity in their larval stages, requiring specific plant species to reproduce, such as the Australian mealybug (*Pseudococcus markharveyi*), which requires Stirling Range Dryandra (*Banksia montana)* (Moir 2021). If the plant is killed by fire, this eliminates the host resource, reducing insect populations and leading to co‐extinction (Moir 2021). Food and habitat specialization is thus an important determinant of insect responses to fire (Mateos et al. 2018).

### 3.8 Cross‐Taxa Patterns

There are some primary commonalities among taxa in how fire shapes populations and the traits that species exhibit (Figure 2). Biota may persist through fire events through two general strategies: in situ survival or movement (Pausas 2019). However, each of these general strategies operates through combinations of traits that may vary between and within major taxonomic lineages.

In situ survival depends on traits that prevent direct mortality (M1). In fire‐prone environments, selection pressures in sessile or less mobile species have resulted in the development of these traits to cope with fire, applicable across taxa (Pausas 2019). There are species that rely on in situ survival and exploit refugia, for example, by burying underground or hiding in logs or deep tree hollows (T2, T3). For example, the Gopher Tortoise ( *Gopherus polyphemus* ) forms deep and extensive burrows that are occupied by other animals during fires (Dziadzio et al. 2016). Essentially, the same strategy operates in the fungus *Pyronema domesticum*, via protection of its spores from fire in the insulated soil profile, primed for post‐fire emergence (Bruns et al. 2020). In addition to the storage of propagules in refugia, plants and fungi, such as the Colombian *Quercus humboldtii*, may survive in situ via intrinsic thermal tolerance of standing individuals and propagules (T1), which do not occur in animal taxa (Aguilar‐Garavito et al. 2023).

Other animal species may exhibit traits that allow them to prevent mortality through ex‐situ movement (M1), such as fire detection and avoidance (T3) and a large dispersal ability (T8). This evasion behavior simultaneously influences the populations of all taxa, including plants and fungi, through the movement of individuals and propagules across the landscape (M2). There are also species from all taxa that benefit from their capacity to disperse large distances (T8) and utilization of a diverse range of habitats, or specifically post‐fire environments (M5, T11), to move to post‐fire landscapes (M3). For example, Western grey kangaroos ( *Macropus fuliginosus* ) travel to enjoy the open and grassy benefits of recently burnt environments (Zylinski et al. 2022). Comparably, Blakely's wattle (*Acacia blakelyi*) is fire‐killed but can disperse widely through the droppings of Western Grey kangaroos and emus, and profit from subsequent germination and establishment in post‐fire environments (Calviño‐Cancela et al. 2008).

Applicable across all taxa and relevant to both in situ and ex situ survival strategies is the suite of traits that determine the capacity of species to persist in different environmental contexts, alter reproduction, and shape interactions with other species. Species deal with predatory or herbivory pressures (M6) via combinations of traits, ranging from utilization of refuges, chemical defenses, and physical defenses to fleeing (T10; Evans and Schmidt 1990). A range of traits related to habitat and resource specialization (T11) is influential across taxa in determining how fire shapes facilitation and the availability of species' required resources and networks (M7). Further, growth rate (T6) and phenology (T7) are important determinants of whether the reproduction of species from all taxa is negatively impacted by when and how frequently fire occurs (M4).

These different strategies influence how fire regimes shape birth, death, and movement, and thus population change. For example, species that possess ex‐situ strategies for coping with fire may experience local population declines post‐fire, but this is not necessarily linked to overall population declines, depending on the spatial configuration of fires across the landscapes they occupy. Local fire intensity and patchiness are, therefore, probably less critical for these species. Still, fire regimes that shape larger landscape processes, such as fire extent and frequency, and overall pyrodiversity will interact with the traits they exhibit to cope with fire (Figure 2). In contrast, species with limited dispersal rely heavily on in situ survival of individuals and their propagules, and so local fire regimes and their spatial granularity will be very important to their population outcomes (Figure 2). This simple characterization of complex fire‐related influences on population processes operating at various scales provides a means of navigating diverse suites of traits across taxa (Figure 2) to identify those critical to vulnerability and resilience under different fire regime scenarios.

## 4 Identifying Traits of Vulnerability and Resilience Under Contemporary Fire Regimes

Fire regimes are shifting globally due to anthropogenic drivers such as climate change and altered land use (Kelly et al. 2023). By linking species traits to fire‐related mechanisms of change, we can identify species that are more likely to be vulnerable or resilient under emerging changes to fire regimes. Combinations of species traits determine the fire regimes to which species populations are most vulnerable or resilient (Batista et al. 2023). The combination of traits influences a species' resilience or vulnerability, irrespective of the taxa or the ecosystem they inhabit.

**FIGURE 3:**
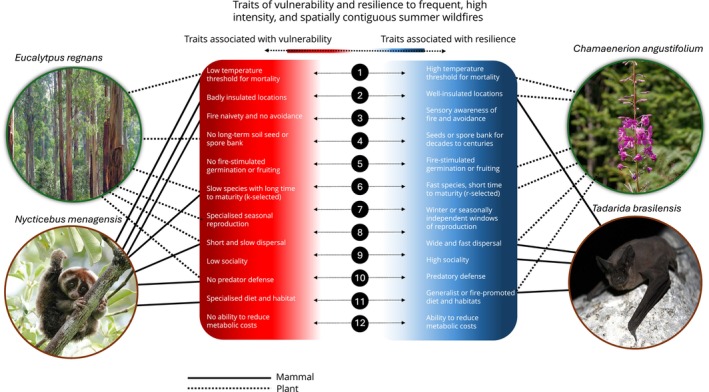
Traits associated with vulnerability and resilience to frequent, high‐intensity, and spatially uniform wildfires. The 12 trait groupings that determine the key mechanisms through which fire influences species populations are expressed with examples of vulnerability (red) or resilience (blue) to high frequency, high‐intensity wildfires with limited patchiness. Four example species from two taxonomic groups (plants, mammals) are displayed, demonstrating how various combinations of these traits may contribute to species' vulnerability or resilience to a specific fire regime. Photo of *Nycticebus menagensis* by Dixon Lau, photo of *Eucalyptus regnans* by David Clode on Unsplash, photo of *Tadarida brasilensis* by USFWS/Ann Froschauer on Wikimedia Commons, photo of *Chamaenerion angustifolium* by H. Zell on Wikimedia Commons.

To illustrate how trait combinations mediate vulnerability or resilience, we chose an exemplar fire regime that is increasing in prevalence under climate change across many environments: high‐frequency, high‐intensity wildfires with low levels of patchiness (Figure 3; Abatzoglou et al. 2018). This fire regime represents a climate‐driven global change scenario that is already contributing to population declines across many taxa in different ecosystems across the world (Kelly et al. 2020). In Figure 3, we present example expressions of the 12 trait groupings identified in Figure 2 that would make a species vulnerable or resilient to this type of fire regime, across taxa and ecosystems. We highlight four example species from two different taxonomic groups and various ecosystems across the world that are inferred to be vulnerable or resilient to high‐frequency, high‐intensity wildfire with limited patchiness, based on the types of traits that they exhibit (Figure 3).

These examples demonstrate how different trait combinations can be used to identify vulnerable or resilient species from different taxonomic groups. For example, fireweed ( *Chamerion angustifolium* ) displays a set of traits that make it resilient to the exemplar fire regime (Pavek 1992). Firstly, it survives fire through resprouting (T1) and protects its root network deep in the soil (T2). It is also highly specialized to the light‐filled and nutrient‐rich environment post‐fire (T11), even preferring the mineral‐rich soils of severe fires (Armour et al. 1984; Pavek 1992). Fireweed also germinates after fire (T5), disperses widely (T8), and reaches reproductive maturity quickly (T6). These traits contribute to a relatively high level of births and immigration and low levels of death and would thus increase local populations under such an exemplary high frequency, high‐intensity, and limited‐patchiness regime. For instance, short‐interval repetition of high‐severity fires can convert Alaskan forests into an herb community dominated by fireweed (Buckley 1958). In contrast, mountain ash ( *Eucalyptus regnans* ) is highly vulnerable to elimination by a high‐frequency, high‐intensity fire regime (McCarthy et al. 1999). This is largely because most individuals die during fire (T1), maturation is slow over multiple decades (T6), and seed dispersal is limited (T8). These traits contribute to a relatively low level of births and immigration and high levels of death (M1), leading to local extinction under the exemplar fire regime (McCarthy et al. 1999). These traits can be applied to other taxa, as we show for two mammal species: the vulnerable Philippine slow loris ( *Nycticebus menagensis* ) and the resilient Mexican free‐tailed bat ( *T. brasiliensis* ) (Figure 3). A useful next step may be to apply this framework to co‐occurring species from various case study ecosystems worldwide.

## 5 Interactions between Fire Related Mechanisms and Other Threatening Processes

**FIGURE 4:**
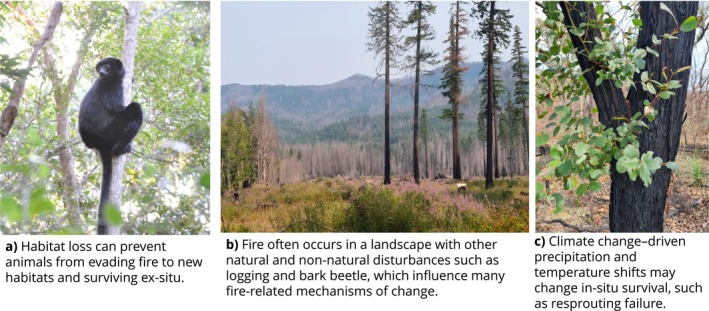
Examples of interactions between other threatening processes and fire‐related mechanisms and traits. (a) Perrier's sifaka, a species threatened by fire due to interactions with habitat loss. Photo by Thomas Martin. (b) A landscape with complex disturbances, Oregon, USA. Photo by Ella Plumanns‐Pouton. (c) Post‐fire traits such as resprouting are also influenced by climate‐driven precipitation and temperature. Photo by Ella Plumanns‐Pouton.

Fire often occurs in a landscape with other threatening processes, such as land‐clearing, post‐fire salvage logging, and species invasions (Figure 4). The interactions between fire regimes and other threatening processes are likely to have large implications for biodiversity conservation but are very poorly understood. Threatening processes may alter fire‐related mechanisms, such as fire evasion, post‐fire immigration, and interspecific processes such as predation, competition, and facilitation‐dependent resources (Souto‐Veiga et al. 2022; Smith et al. 2016). While many species exhibit traits that confer resilience to particular fire regimes, interacting threatening processes may reduce the efficacy of traits as a strategy for persistence under certain fire regimes. For example, the Perrier's sifaka ( *Propithecus perrieri* ) is an arboreal prosimian that relies on the tropical tree canopy for its habitat in Madagascar (Salmona et al. 2015). It possesses characteristics that should make it capable of evading fire and emigrating elsewhere, such as large body mass, wide dispersal, and dietary plasticity. However, extensive land‐clearing has restricted Perrier's sifaka's population to three isolated forest patches totaling 200km^2^ (Salmona et al. 2015), severely limiting its dispersal ability, a key trait for coping with fire (Figure 4a). Habitat loss, fragmentation, or conversely afforestation (tree planting) may increase the difficulty of moving through other land types, preventing species from utilizing dispersal to evade fire and find new habitats (Driscoll et al. 2021; Figure 4b). Other threatening processes can thus “switch off” traits that determine the mechanism through which fire influences species.

Climate change is also likely to influence the expression of traits and thus determine the mechanisms through which fire may threaten population persistence. Antecedent and post‐fire precipitation and temperature are key influences on the ability of plant species to resprout (Cruz et al. 2003; Karavani et al. 2018), germinate and recruit post‐fire (Richards and Lamont 1996), and accumulate a seedbank (Enright et al. 2015). The full impacts of how changing climates may affect these key fire‐related plant processes are unclear, but they are likely to change how plant species respond to fire regimes (Henzler et al. 2018; Nolan et al. 2021; Figure 4c). Changes to plant populations via these mechanisms are also likely to impact animals via their habitat and diet (Araújo and Rahbek 2006). Climate change may also directly impact animal species and the mechanisms through which fire influences them (Radchuk et al. 2019). For example, climate change can alter metabolic costs (Briscoe et al. 2023) and phenology (Cohen et al. 2018), potentially altering species' ability to cope with climate‐driven fire regime changes.

## 6 Opportunities for Further Research and Utility for Conservation

Here, we have synthesized a rich body of work on fire‐related traits and mechanisms, described independently across studies that concern individual species or taxa (Driscoll et al. 2010), with the aim of identifying those shared between species across multiple taxonomic groups. By defining four direct and four indirect mechanisms of fire‐related population change and the traits that underpin these responses, this framework advances the evidence base for how fire impacts terrestrial biota. This framework will help inform predictions of population trajectories for diverse taxa in the context of globally changing fire regimes (Figure 3). It may, therefore, contribute to developing more integrated management interventions that are needed to promote population persistence for species across multiple taxa.

A cross‐taxa approach can support management strategies for biodiversity conservation by pinpointing mechanisms of fire responses and relevant traits that may apply to a variety of species. This will help identify the specific fire regimes that threaten multiple species' persistence and require mitigation action in the context of other threats and decision‐making needs (DAWE 2022). For example, further research could, with the support of research that documents individual species ecology, use this framework to identify key species likely to be impacted by certain kinds of regimes in the context of other landscape and climatic changes and why. This would help guide decision‐making based on the sensitivity of multiple species, such as those most sensitive to particular fire regimes, and identify trade‐offs between species that may exhibit divergent responses (Keith et al. 2002).

The present framework also helps identify interactions between species, such as facilitation, competition, or predation (mechanisms 4–8), with the potential to inform decision‐making and management interventions that account for these dependencies. In this way, the present framework should help scale up species‐specific responses across taxa to elucidate fire‐related risks at the ecosystem level and support fire‐management decisions. An important next step will be applying and testing the use of such a framework across taxa in a variety of different ecosystems and regions, with the support of trait information, and in the context of the needs of decision makers and decision science approaches (Rose et al. 2019).

Here, we focused on providing a cross‐taxa framework of the key mechanisms of fire‐related population change and the traits that influence these mechanisms. Applying such an approach to a generalized suite of species requires good evidence on the types of traits species possess. While we have identified many traits that we predict to be important, these are supported by varying levels of evidence. Recent efforts to collate trait databases have enormous scientific and conservation potential (Kattge et al. 2020), but for many species, knowledge on traits is still developing (Etard et al. 2020; Tyler et al. 2012). This is particularly true for understudied taxa, such as amphibians and invertebrates, and especially for non‐insect invertebrates (Jolly et al. 2022). In Australia, for example, as of 2021, just six of the 30 invertebrate phyla are featured in peer‐reviewed literature pertaining to fire (Saunders et al. 2021). Working to identify and fill knowledge gaps on species traits will be important to protect against population declines in the face of global change (Plumanns‐Pouton, Swan, et al. 2024a). It will be important to build knowledge on the limits of species traits to shifting fire regimes and other environmental drivers. This also emphasizes that a generalized framework is not a substitute for species‐specific understanding but a means of improving and extending how species‐specific approaches are used in designing fire‐management strategies for biodiversity conservation. Research approaches that relate to species, taxonomic groups, generalized traits, and entire ecosystems are needed to protect biodiversity under contemporary fire regimes (Driscoll et al. 2010; Driscoll et al. 2024).

Our focus on population‐level processes provides an essential foundation for building a framework that addresses higher levels of ecological organization that involve interactions among fire‐response mechanisms. While we have focused on taxonomic groups and the traits that specific species possess, further work is needed to scale up fire‐related processes to ecosystem‐level changes. We expect work that seeks to identify interactions between taxa, including trophic groups, and their interactions with other ecosystem processes will be crucial to conserving biodiversity in the face of global change (Charles et al. 2025). Further research should work to identify the traits most likely to be impacted under future climates and landscape contexts and how this may affect population persistence. Empirical work is needed to quantify the limits of these traits in responding to different aspects of the fire regime, climate, habitat loss, and fragmentation (Buhk et al. 2007; Driscoll et al. 2021). We have only considered terrestrial eukaryotes and so an investigation of microbial, marine, and freshwater biota is also warranted, particularly given the potentially extreme fire regime effects on freshwater fish and macroinvertebrates (Gomez Isaza et al. 2022).

## 7 Conclusions

As fire regimes change and interact with other shifting abiotic drivers, we require integrative tools to identify fire‐related threats to species. For the first time, this framework synthesizes fire‐related mechanisms of population change and the traits on which these depend across much of the taxonomic spectrum of terrestrial macrobiota and fungi. It shows strong commonality and some key differences in mechanisms and traits across taxa. Because this framework identifies key traits that determine fire‐related population change, we reason that it, alongside trait data, can provide a means to identify vulnerable and resilient species across taxa. Further, the framework highlights the contexts (i.e., taxa and fire regimes) in which in situ survival, in situ reproduction, or, alternatively, dispersal and colonization, are each critical in population persistence under recurring fires. These generalized strategies will be a key foundation for broad‐based fire management for biodiversity conservation.

## Author Contributions

**Ella Plumanns‐Pouton:** conceptualization, investigation, project administration, visualization, writing – original draft, writing – review and editing. **Julianna L. Santos:** conceptualization, investigation, visualization, writing – original draft, writing – review and editing. **Cristina Aponte:** conceptualization, investigation, writing – original draft, writing – review and editing. **Lluís Brotons:** conceptualization, investigation, writing – original draft, writing – review and editing. **Luke T. Kelly:** conceptualization, investigation, writing – original draft, writing – review and editing. **Stephen C. Mason Jr.:** conceptualization, investigation, writing – original draft, writing – review and editing. **Kirsten Parris:** conceptualization, investigation, writing – original draft, writing – review and editing. **Lauren Ponisio:** conceptualization, investigation, writing – original draft, writing – review and editing. **David A. Keith:** conceptualization, investigation, supervision, writing – original draft, writing – review and editing.

## Conflicts of Interest

The authors declare no conflicts of interest.

## Supporting information


**Data S1:** Supporting Information.

## Data Availability

Data sharing not applicable to this article as no datasets were generated or analyzed during the current study.
