# Supplementary material for: The Mechanisms Through Which Fire Drives Population Change in Terrestrial Biota
Source: Glob Chang Biol. 2025 Sep 22;31(9):e70479. doi: 10.1111/gcb.70479 (PMC12451515; doi:10.1111/gcb.70479)
Supplement: Supplementary file 1 — Data S1: Supporting Information. [file GCB-31-e70479-s001.pdf]

Table S1. A proposed framework of the generalised mechanisms through which fire influences terrestrial populations. Rows list eight proposed key mechanisms through which fire influences population change in terrestrial biota, via impacts on individuals' birth, death, and movement, and via community-driven changes that indirectly influence demographic processes. Each column refers to a different taxon. An '+' indicates that this mechanism is expressed by several species within a taxon; An '?' indicates that it is unknown if it is expressed by several species within a taxon. When there are two mechanisms (promotion and disruption), '+' indicates the promotive mechanism is expressed by several species within a taxon, and '-' indicates the disruptive mechanism is expressed by several species within the taxon' and '+/-' indicates both the promotion and disruption mechanisms are expressed by several species within the taxon. Timescale refers to the period in which the mechanism can occur.

Mechanism	Timescale	Fungi	Plants	Mammals	Birds	Reptiles	Amphibians	Insects
Primarily individual-based mechanisms								
1. Direct mortality (vs. survival)	During fire-several years after fire	+	+	+	+	+	+	+
2. Fire evasion induced emigration	During fire or shortly after fire	?	?	+	+	+	+	+
3. Fire-mediated immigration	During fire-weeks after fire	+/-	+/-	+/-	+/-	+/-	?	+/-
4. Fire-mediated reproduction	During fire-several years after fire	+/-	+/-	-	-	-	-	-
Primarily community-facilitated mechanisms								
5. Fire mediated facilitation	Days - centuries after fire	+/-	+/-	+/-	+/-	+/-	+/-	+/-
6. Fire-promoted predation	During fire – centuries after fire	+	+	+	+	+	+	+
7. Fire-released interspecific competition	Days - centuries after fire	+	+	+	?	?	?	+
8. Fire-mediated susceptibility to disease and parasitism	Days - centuries after fire	?	+/-	-	?	-	-	?

Table S2. Examples of literature related to each mechanism for each taxa. Literature are all cited in the main text and the full citation can be found in the bibliography.

Mechanism	Fungi	Plants	Mammals	Birds	Reptiles	Amphibians	Insects
**Direct mortality (vs survival)**	Reazin et al. 2016	Whelan & Ayre 2022	Robinson et al., 2013	Jolly et al., 2022	Shine et al., 2016	Pilliod et al., 2003	Dole et al., 2023
**Fire evasion induced emigration**	? Camacho et al., 2018	? Monty et al., 2013	Pruetz & LaDuke, 2010	Brotons et al., 2012	Nimmo et al., 2019	Pilliod et al., 2003	Liu et al., 2022
**Fire-mediated immigration**	Horton, 2017	Benedicto-Royuela et al., 2024	Archibald et al., 2015	Bonta et al., 2017	Smith, 2018	NA	Saint-Germain et al., 2008
**Fire-mediated reproduction**	Hughes et al., 2020;	Lamont & Downes, 2011	Griffiths & Brook 2015	Murphy et al., 2010	Weiss & Brower, 2021	Muñoz et al., 2019	Decker & Harmon-Threatt, 2019
**Fire mediated facilitation**	Chungu et al., 2020	Nuland et al., 2013	Westlake et al., 2020	Hovick et al., 2017	X.Santos et al., 2014	Hromada et al., 2018	Bargmann et al., 2016
**Fire-promoted predation**	Luo & Fox, 1994	Giljohann et al., 2017	Hradsky et al., 2017	Churchwell et al., 2008	Wilgers & Horne, 2007	McGregor et al., 2017	Rocha et al., 2008
**Fire-released interspecific competition**	Carlsson et al., 2014;	Keith & Bradstock, 1994	Allen et al., 2022	NA	NA	NA	Burkle et al., 2019
**Fire-mediated susceptibility to disease and parasitism**	NA	N. Moore et al., 2014	Donaldson et al., 2023	NA	Álvarez-Ruiz et al., 2021	Kaiser et al., 2021	NA

Table S3. Examples of literature related to each trait for each taxa.

Traits	Fungi	Plants	Mammals	Birds	Reptiles	Amphibians	Insects
**T1. Insulative and regenerative tissues of individuals and propagules**	Baar et al., 1999	Pausas 2015 Clarke et al., 2013	NA	NA	NA	NA	NA
**T2. Location of host, nest or refugia**	Cairney & Bastias, 2007	Pausas et al., 2018	Culhane et al., 2022	Shine et al., 2016	Jordaan et al., 2020	Penman et al., 2006	Cane & Neff, 2011
**T3. Fire detection and avoidance behaviours**	NA	NA	Pruetz & LaDuke, 2010	Engstrom, 2010	Álvarez-Ruiz, Belliure, & Pausas, 2021	Grafe et al., 2002	Evans 1971
**T4. Propagule longevity**	Nara 2009	Plumanns Pouton et al., 2024C	NA	NA	NA	NA	NA
**T5. Fire stimulated germination and fruiting**	Bruns et al., 2019	D. T. Bell, 1999	NA	NA	NA	NA	NA
**T6. Growth rate (incl. regeneration tissues) and fecundity**	Whitman et al., 2019	Zedler, 1995	Tokushima & Jarman, 2017	Woinarski & Recher, 1997	Shine et al., 2016	Mahony et al., 2022	Brown et al., 2017
**T7. Phenology**	Andrew et al., 2018	Beck et al., 2023	Begg et al., 1981	Dale et al 2005	Means & Campbell, 1981	Muñoz et al., 2019	Sandoval, 2000
**T8. Dispersal ability**	Chaudhary et al., 2022	Ahler et al., 2023	Forney & Peacock, 2024	Brotons et al., 2005a	(Ferreira et al., 2019).	Hossack & Pilliod 2011	Koltz et al., 2018
**T9. Sociality**	NA	NA	Hollén & Radford, 2009	Downing et al., 2020	NA	NA	Glasier et al., 2015

Traits	Fungi	Plants	Mammals	Birds	Reptiles	Amphibians	Insects
**T10. Predator defence**	Kunzler et al., 2018	Barton & Koricheva, 2010	Nimmo et al. 2021	Lima 2009	Lillywhite et al., 1977	Murray et al., 2004	Evans & Schmidt, 1990
**T11. Resource and habitat specialisation**	Certini et al., 2021	Plumanns Pouton et al., 2024a	Dickman & Happold, 2022	Rainsford et al., 2023	Smith, 2018	Hossack et al., 2009	Arnan et al., 2013
**T12. Ability to induce Torpor**	NA	NA	Geiser et al., 2018	Schleucher 2004	Fenner & Bull, 2007	NA	NA
